# Psychiatry Trainees’ Perspectives on Psychotherapy Training in Residencies Worldwide

**DOI:** 10.1192/j.eurpsy.2024.1659

**Published:** 2024-08-27

**Authors:** R. M. Salgado, O. von Doellinger

**Affiliations:** ^1^Centro Hospitalar do Tâmega e Sousa, Penafiel; ^2^Faculty of Medicine of the University of Porto, Porto, Portugal

## Abstract

**Introduction:**

Incorporating psychotherapy into the curricula of psychiatry residency programs has been proven difficult, even in countries where psychotherapy training is a requirement for psychiatry residents to become psychiatrists. There is a risk that future psychiatrists lacking psychotherapy skills will be restricted in managing the wide scope of disorders and personalities they will face in clinical practice. It is important to assess what psychiatry trainees around the globe have to say about psychotherapy training as part of their residency curricula.

**Objectives:**

The primary purpose of the article was to assess psychiatry trainees’ perspectives on psychotherapy training in residency programs worldwide.

**Methods:**

The authors performed a narrative review, resulting 19 original research studies, published between 2001 and 2021, evaluating psychiatry residents’ perspectives by the application of a questionnaire.

**Results:**

Nineteen articles were included in this review. Most of the studies were developed across European countries (47.4%) and in the USA (36.8%). Psychiatry residents are interested in and value psychotherapy training, and some consider it should be an obligatory competency for psychiatrists, as it already occurs in some countries worldwide. Even though, most psychiatry trainees feel dissatisfaction with the existing training in residency curricula, pointing out concerns related to the quality of resources such as courses of psychotherapy and supervision of cases, time within the residency period, and financial constraints. In terms of personal psychotherapy, we found contrasting views of its importance in psychotherapy training for psychiatry residents. A crucial finding was that psychiatry residents tend to lose interest in psychotherapy during the years of the residency, and dissatisfaction with the quality of the psychotherapy curricula, lack of support, and low self-perceived competence in psychotherapy by trainees were factors associated with reduced interest in psychotherapy training.

**Conclusions:**

At a time when psychotherapy is increasingly becoming acknowledged to play a central role in the treatment of most psychiatric disorders, current training is failing to provide these competencies to psychiatry trainees. Serious reflection must be given to both the extent of the guidelines and the practical opportunities for psychotherapy training so future psychiatrists can be qualified to provide an accurate biopsychosocial model of psychiatric care. The authors postulate that maintaining residents’ interest in psychotherapy requires improvements in the residency curricula and departmental leadership must support trainees’ goals of becoming comprehensively trained psychiatrists.

**Disclosure of Interest:**

None Declared

